# Breast Gangrene

**DOI:** 10.1186/1749-7922-6-29

**Published:** 2011-08-19

**Authors:** Imtiaz Wani, Iftikhar Bakshi, Fazl Q Parray, Ajaz A Malik, Rauf A Wani, Mubashir Shah, Irfan Husasin, Altaf Malik, Sajad Wani, Wahid Syed

**Affiliations:** 1 Department of General Surgery, Sheri-Kashmir Institute of Medical Sciences, Srinagar, Kashmir, India

**Keywords:** Gangrene, breast, diabetes, belladonna, lactation

## Abstract

**Background:**

Breast gangrene is rare in surgical practice. Gangrene of breast can be idiopathic or secondary to some causative factor. Antibiotics and debridement are used for management. Acute inflammatory infiltrate, severe necrosis of breast tissue, necrotizing arteritis, and venous thrombosis is observed on histopathology. The aim of was to study patients who had breast gangrene.

**Methods:**

A prospective study of 10 patients who had breast gangrene over a period of 6 years were analyzed

**Results:**

All the patients in the study group were female. Total of 10 patients were encountered who had breast gangrene. Six patients presented with breast gangrene on the right breast whereas four had on left breast. Out of 10 patients, three had breast abscess after teeth bite followed by gangrene, one had iatrogenic trauma by needle aspiration of erythematous area of breast under septic conditions. Four had history of application of belladonna on cutaneous breast abscess and had then gangrene. All were lactating female. Amongst the rest two were elderly, one of which was a diabetic who had gangrene of breast and had no application of belladonna. All except one had debridement under cover of broad spectrum antibiotics. Three patients had grafting to cover the raw area.

**Conclusion:**

Breast gangrene occurs rarely. Etiology is variable and mutifactorial. Teeth bite while lactation and the iatrogenic trauma by needle aspiration of breast abscess under unsterlised conditions could be causative. Uncontrolled diabetes can be one more causative factor for the breast gangrene. Belladonna application as a topical agent could be inciting factor. Sometimes gangrene of breast can be idiopathic. Treatment is antibiotics and debridement.

## Introduction

Gangrene of breast is rare to see [1]. There are only few cases of breast gangrene reported in the literature. This is regarded as cosmetic blemish and is agony for the female. Gangrene of breast can be idiopathic or occurs after some secondary to some causative agent. Occurrence of breast gangrene in the diabetes, after application of a topical agent or of idiopathic cause is scarcely reported in literature. Its medico-surgical management is an emergency [2]. Treatment involves debridement, antibiotics and sometimes mastectomy. The aim was to study clinical presentation and management of patients with breast gangrene.

## Methods

A study of 10 female patients who presented with the breast gangrene from 2005 to 2011 was done at Sheri-Kashmir Institute of Medical Sciences. Age, site, size, treatment and surgical procedures were studied.

## Results

**Figure 1:**
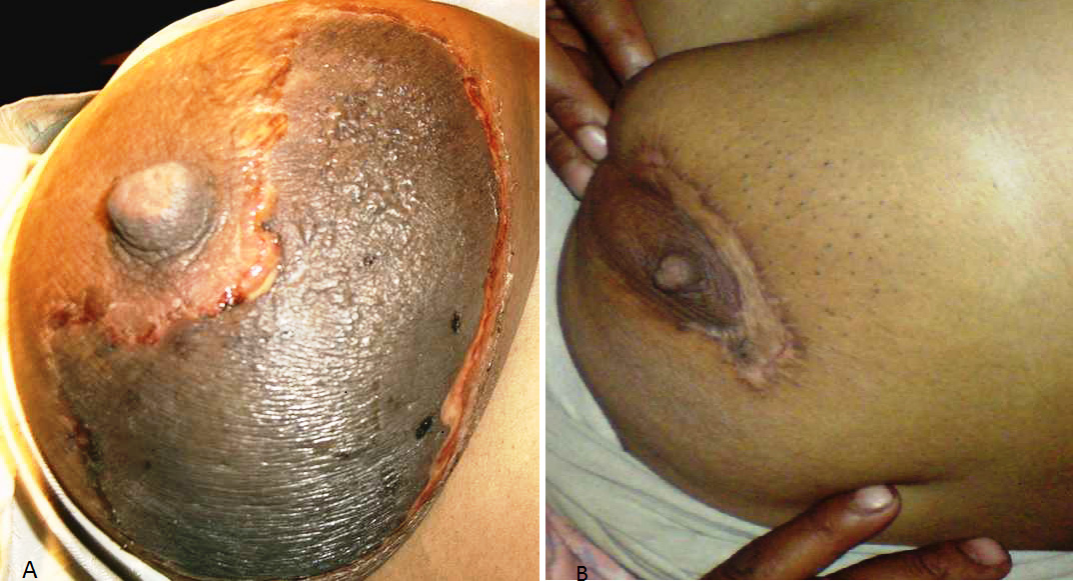
**(A) Gangrene breast after application of belladonna paste in a lactating female; (B)**:** Breast after debridement and grafting.**

**Figure 2:**
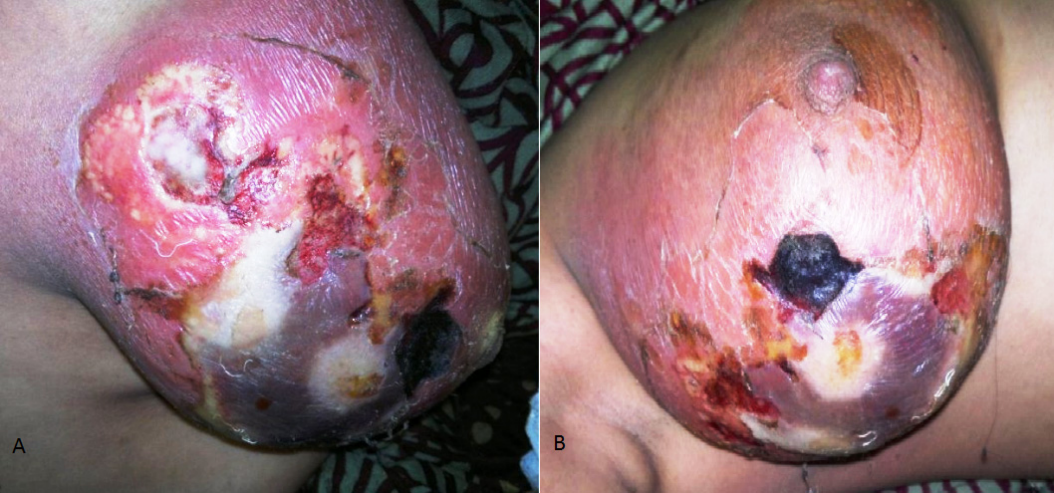
**(A) Gangrene of breast following tooth bite in a lactating female; (B) Typical gangrene patch on breast following tooth bite by infant in lactating female.**

**Figure 3:**
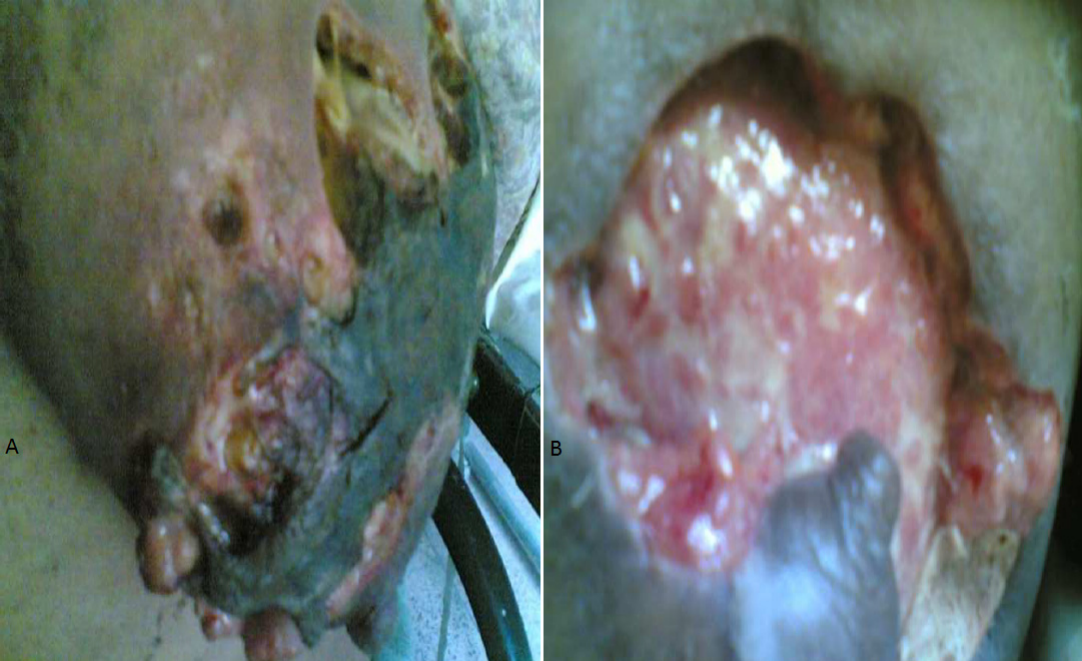
**(A) Gangrene in a breast after she had needle aspiration for confirmation of pus and progressed to necrotizing fascitis in a lactating female; (B) Breast after serial debridements**.

**Figure 4:**
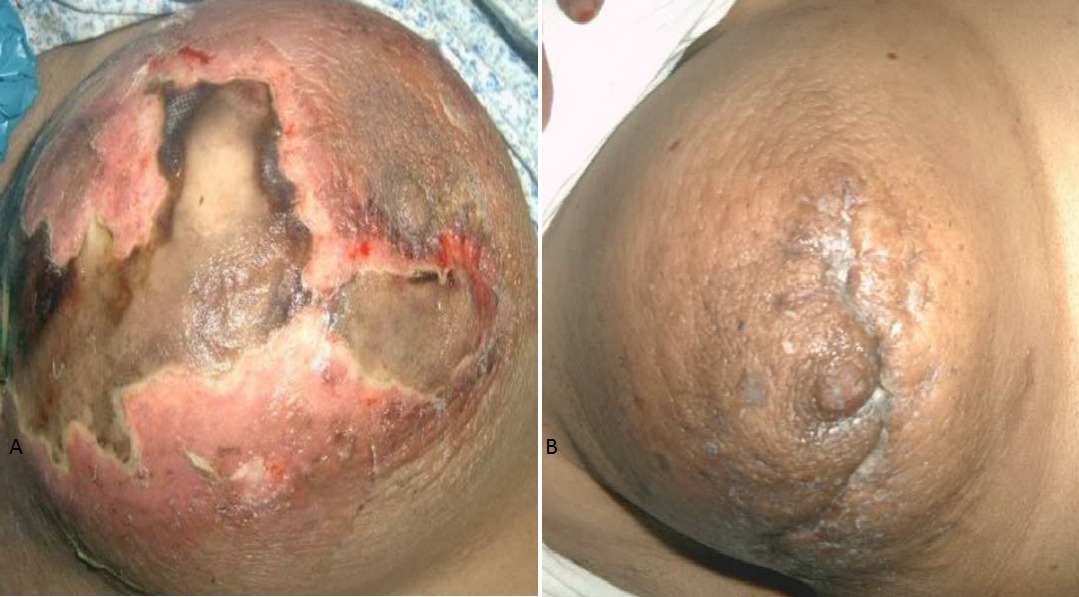
**(A) Gangrene of breast in diabetic female which progressed to necroting fascitis; (B) Breast after control of blood sugar and serial debridements.**

**Figure 5:**
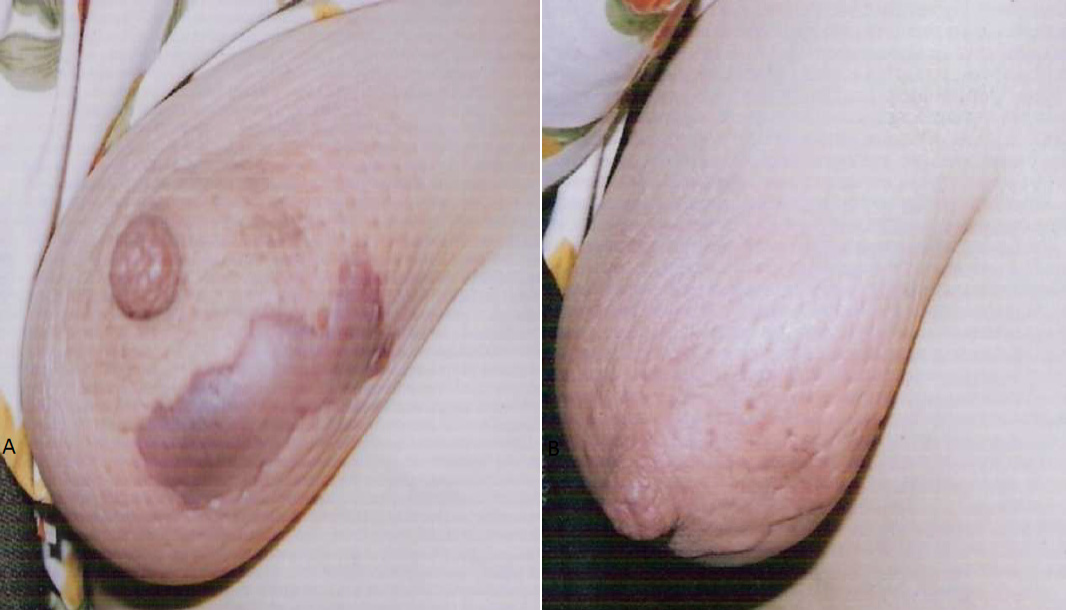
**(A) Gangrene of breast in an elderly female of idiopathic cause; (B) Breast after antibiotic treatment with no debridement.**

Total of 10 patients were studied. In study group, six patients had gangrene on right breast, while four had gangrene on left breast. Age ranged form 23 years to 64 year old female.8 were lactating child, lactation period varied form 3 weeks to 7 months period. In lactating group, 2 females were primiparous and 6 were multiparous. One was an elderly diabetic aged 58 years and one was a non diabetic old lady aged 64 years. Prior lactational mastitis and with subsequent breast gangrene was present in 8 cases (Figure 1A, 2A, 3A), out of which 3 patients had the teeth bite by baby only while lactation (Figure 2A). One had iatrogenic trauma by needle aspiration of erythematous area of breast under unsterilised conditions (Figure 3A). Among females with breast gangrene, two females had a gangrene of breast in a puerperal period; both had no documentation of any puerperal sepsis. Two elderly female had breast abscess before onset of gangrene. (Figure 4A, 5A).

Four patients had local application of a belladonna paste on a mastitis area of the breast had time interval from application of a topical agent to appearance of gangrene varied form 48 hours to 96 hours. (Figure 1A) Diabetic patient who had breast gangrene had no history of application of any topical agent, gangrene appeared 120 hours after appearance of breast abscess (Figure 4A). Non diabetic elderly female having idiopathic breast gangrene had gangrene after 48 hours of mastitis (Figure 5A). All had skin and subcutaneous gangrene. Size of lesion varied from small localized gangrene patch to diffuse involvement, nipple areola complex was spared in all cases. Whereas two patients had extensive involvement of mammary tissue and fatty tissue involvement with systemic toxicity progressed to necrotizing fascitis of breast. Of these one was diabetic and another was a lactating female. (Figure 3A, 4A) No axillary lymphadenopathy was present in any case. All had the broad spectrum antibiotics started at the time of admission in hospital after taking wound and blood culture. Impinem-cilastatin vancomycin was used was used in all the patients.

Wound cultures in cases who had teeth bite and in diabetic revealed heavy growth of styphalcoccus aureus showing sensitivity to linzeolid, Methicillin and Vancomycin. Wound culuture from other patients had polymicrobial skin flora (E.Coli, Bacteroids, Proteus, Enterococcus and anaerobic streptococci ) in all cases. Blood culture yield grew E.Coli in diabetic female whereas all other patients had sterile blood culture.

Debridement was done in 9 cases; three had grafting, one had graft rejection and refused the second grafting (Figure 1B &2B). Diabetic patient who had uncontrolled diabetes was managed by insulin. Multiple serial debridements were done in 3 patients (Figure 2B, 3B &4B). One case, elderly female who had idiopathic breast gangrene, was managed conservatively with broad spectrum antibiotics required no debridement.(Figure 5B). Histopathology of debridement tissue showed features of breast abscess and necrosis, inflammatory infiltrate with thrombosis of vessels.

## Discussion

Breast gangrene is rarely seen in surgical practice [1]. The rarity of a gangrene of the breast is attested by the fact that this entity is not mentioned in most of the recent textbooks or monographs on diseases of the breast [3]. The occurrence of such an unusual complication of diabetes as gangrene of the breast, seems worth reporting [4]. The nature of this entity is obscure and remains to be uninvestigated and undiscovered. Breast gangrene is considered as Fournier type of gangrene caused by massive fulminating type of infection complicated by obiliterative arteritis. Gangrene of breast is usually a unilateral affection, and rarely can occur in both breasts. Preceding mammary mastitis or breast abscess or without any mastitis, is seen before occurrence of gangrene. Type of necrosis in gangrene of breast is a coagulative necrosis or dry type of necrosis. Breast gangrene is well reported with use of anticoagulant therapy, trauma, thrombophlebitis, puerperal sepsis, pregnancy, lactation, diabetes mellitus, beta hemolytic streptococci infection, or carbon monoxide poisoning are other causes which can incite gangrene of breast [1,4-8]. Recently there has been seen reported in HIV infection [9]. Sometimes they can be idiopathic or, after taking core biopsy of breast or can occur after surgery [10]. In idiopathic form, the initial manifestation is mammary pain with no antecedent history of trauma or infection and patient develops well recognized area of skin which may develop a peau'd orange appearance. A spontaneous occurrence of breast gangrene of unknown etiology was reported by Cutter in his case of apoplexy of breast [11]. Spontaneous infarction of physiologically hyperplasic breast tissue with sparing of overlying skin mimicking as breast tumor has been reported to occur in pregnancy and lactation [12,13].

There was no oral contraceptive intake or any other significant drug ingestion, or any evidence of thromboembolic events present in any patient. In this series there was history of trauma in form of teeth bite in 3 patients and iatrogenic trauma with syringe which was dry tap under septic conditions for confirmation of pus in erythematous area of breast. Application of belladonna paste on erythematous area of breast was seen in 4 patients. Gangrene of breast in the diabetes is recognized as a grave complication^4^. In diabetes, hyperglycemia, risk for infection and increased vascular atherosclerosis contributes to the increased susceptibility to gangrene.

A sequence of events seen is that after start of mammary mastitis with or without topical application of topical belladonna was there and a black ecchymosis of the dermal abscess is observed. This necrosis is always starts in skin and more on peripheral parts of mastitis area or breast abscess. Time of appearance of gangrene varies from 48-96 hours in who had start of gangrene after application of topical agent. Diabetic patient had appearance after 120 hours after start of dermal abscess. After the initiation of this dermal gangrene, there is spread of this gangrene in all directions of restricted to cutaneous abscess and frequently rapidly evolves into black patch. A full eschar forms at the end. Sometimes the gangrene progresses into underlying tissue of breast of fat lobules and glandular tissue presenting as necrotizing fasciitis. In non diabetic, 48 hours after mastitis had appearance of gangrene. Apparently no history of any inciting factor was present and was managed on broad spectrum antibiotics without any debridement.

There are reports where belladonna extract was applied on threatened milk abscess and patient had recovery [14]. This drug has been ascertained to possess galactifuge properties; and accordingly, being applied in the form of extract or ointment around the nipple in these cases, it speedily checks the secretion of milk, and with it the inflammation. This is to be stressed that in far rural areas with no easy access to medical facilities, there still used be topical application of belladonna paste in mammary abscess and but all do not get gangrene and have well resolution. This aspect cannot suggest belladonna is precipitating factor for breast gangrene. Variations to cutaneous response and hypersensitivity to belladonna application could be in some cohorts could be precipitating factor.

An evidence of widespread venous occlusions documented histologically had been reported in majority of cases of breast infarction associated with a nonspecific panarteritis, focal endarteritis obliterans, and inflammation of small veins [13]. Microthrombi are often causes of this necrosis [15]. The extensive thrombosis evident in the subcutaneous vessels in breast gangrene suggests that the administered antibiotics does not reach the infected regions in sufficient quantity to be effective in diabetic breast gangrne [16]. In hemorrhagic type mammary gangrene once gross tissue necrosis or secondary infection ensue, the biopsy becomes non-specific and non-diagnostic and there is a distinct lack of arteriolar thrombosis and no evidence of vascular or perivascular inflammation in comparison to mammary gangrene after mastitis where there is both vessel thrombosis and evidence of inflammatory infiltrate. Mixed anaerobic and aerobic florae are often responsible for the infection in gas gangrene of breast [2].

Successful surgical outcome is usually expected secondary to expeditious surgical intervention in the form of wide local excision of the gangrenous breast with proper toileting tissue along with broad-spectrum antibiotics followed by reconstructive procedures. Serial debridements are required in some patients where there is diffuse involvement. Grafting is done where there is large deficit Sometimes mastectomy is mandatory in extensive involvement

## Conclusion

Gangrene of breast is rare and ignorance on part of patient contributed to this malady. Application of topical agent of belladonna on cutaneous abscess in lactational female could be aggravating factor. In uncontrolled diabetes breast abscess has propensity for progression to gangrene. Sometimes gangrene of breast can be of idiopathic cause. Debridement continues to be gold standard in gangrene of breast.

## Consent

'Written informed consent was obtained from the patient for publication of the manuscript and accompanying images. A copy of the written consent is available for review by the Editor-in-Chief of this journal'

## Competing interests

The authors declare that they have no competing interests.

## Authors' contributions

designed the study, contributed in literature search, data analysis, manuscript writing. IB, FP, AM and RW helped in study design, data analysis, manuscript writing and editing. MS, IH, AM SW and WS participated in study design, supervised the write up of the manuscript and edited the manuscript before submission. All the authors read and approved the final manuscript

## List of Abbreviations

None
